# The Role of Extracorporeal Membrane Oxygenation ECMO in Accidental Hypothermia and Rewarming in Out-of-Hospital Cardiac Arrest Patients—A Literature Review

**DOI:** 10.3390/jcm12216730

**Published:** 2023-10-25

**Authors:** Hubert Hymczak, Aleksandra Gołąb, Sylweriusz Kosiński, Paweł Podsiadło, Dorota Sobczyk, Rafał Drwiła, Bogusław Kapelak, Tomasz Darocha, Dariusz Plicner

**Affiliations:** 1 Department of Anesthesiology and Intensive Care, St. John Paul II Hospital, 31-202 Krakow, Poland; h.hymczak@szpitaljp2.krakow.pl (H.H.); r.drwila@szpitaljp2.krakow.pl (R.D.); 2 Faculty of Medicine and Health Sciences, Andrzej Frycz Modrzewski Krakow University, 30-705 Krakow, Poland; 3 Faculty of Medicine and Dentistry, Pomeranian Medical University in Szczecin, 70-204 Szczecin, Poland; 4 Center for Research and Innovative Technology, John Paul II Hospital, 31-202 Krakow, Poland; 5 Department of Interdisciplinary Intensive Care, Jagiellonian University Medical College, 31-008 Krakow, Poland; sylweriusz.kosinski@uj.edu.pl; 6 Institute of Medical Sciences, Jan Kochanowski University, 25-369 Kielce, Poland; p.podsiadlo.01@gmail.com; 7 Department of Cardiovascular Diseases, John Paul II Hospital, 31-202 Krakow, Poland; dorotasobczyk@yahoo.com; 8 Department of Cardiovascular Surgery and Transplantation, John Paul II Hospital, 31-202 Krakow, Poland; bogus.kapelak@gmail.com (B.K.); plicner.dariusz@gmail.com (D.P.); 9 Jagiellonian University Medical College, 31-008 Krakow, Poland; 10 Department of Anesthesiology and Intensive Care, Medical University of Silesia, 40-055 Katowice, Poland; 11 Unit of Experimental Cardiology and Cardiac Surgery, Faculty of Medicine and Health Sciences, Andrzej Frycz Modrzewski Krakow University, 30-705 Krakow, Poland

**Keywords:** accidental hypothermia, cardiac arrest, extracorporeal membrane oxygenation, hypothermia

## Abstract

Accidental hypothermia, defined as an unintentional drop of the body core temperature below 35 °C, is one of the causes of cardiocirculatory instability and reversible cardiac arrest. Currently, extracorporeal life support (ECLS) rewarming is recommended as a first-line treatment for hypothermic cardiac arrest patients. The aim of the ECLS rewarming is not only rapid normalization of core temperature but also maintenance of adequate organ perfusion. Veno-arterial extracorporeal membrane oxygenation (ECMO) is a preferred technique due to its lower anticoagulation requirements and potential to prolong circulatory support. Although highly efficient, ECMO is acknowledged as an invasive treatment option, requiring experienced medical personnel and is associated with the risk of serious complications. In this review, we aimed to discuss the clinical aspects of ECMO management in severely hypothermic cardiac arrest patients.

## 1. Introduction

Hypothermia is defined as a clinical condition in which the body core temperature falls below 35 °C. “Tc” will be used henceforth to denote “body core temperature”. According to etiology, hypothermia can be classified as unintentional (accidental), i.e., from exposure to cold, and intentional (therapeutic). The so-called therapeutic hypothermia, which means a targeted and controlled reduction in Tc, is used in cardiological, neurosurgical and cardiosurgical procedures or in intensive care units [1,2]. It has been established that this procedure improves survival and outcomes from some clinical conditions, including in out-of-hospital cardiac arrest survivors, mostly due to its neuroprotective effect and a decrease in the risk of encephalopathy [1]. However, the optimal target temperature management in this group of patients remains unknown. It has been established that lower temperature, i.e., below 34 °C, does not improve survival nor neurological outcome after normothermic cardiac arrest and increases the risk of arrythmias compared to mild hypothermia and normothermia [3,4,5,6]. It should be also emphasized that avoiding fever seems to be a more important issue [5,6]. In contrast, the survival after cardiac arrest, induced by hypothermia is higher compared to normothermic cases, which is related to a decrease in brain oxygen requirements. Nonetheless, in accidental, uncontrolled development of hypothermia, vital signs decrease and finally this condition may lead to death [2]. Some factors, including degree and cooling time, concomitant diseases and the availability of rewarming techniques influence the prognosis of accidental hypothermia [7].

Currently, in severely hypothermic cardiac arrest (H-CA) patients, extracorporeal life support (ECLS) is recommended [8,9]. Veno-arterial extracorporeal membrane oxygenation (VA-ECMO) is a preferred technique, mainly due to its low anticoagulation requirements (or even omitting anticoagulation is possible because hypothermia itself impairs the coagulation process) and potential to prolong circulatory support [8,10]. Moreover, ECMO allows to control rewarming, avoiding the issue of a rapid increase in Tc [11]. Despite the fact that ECMO is regarded as the most effective treatment of H-CA patients, it is also a highly invasive technique, that is associated with an increased risk of complications, such as bleeding, vessel injury, thromboembolic events, limb ischemia or infections. Additionally, implantation and surveillance of ECMO require experienced medical personnel [12]. Recent studies, which investigated the effectiveness of ECMO use in H-CA patients, are not robust and yielded inconsistent conclusions [13,14,15]. Our previous report showed that patients with severe accidental hypothermia with circulatory instability can benefit from ECMO rewarming without an increased risk of complications, which is in line with the latest meta-analysis by Low et al. [13,14]. They indicated the advantage of ECLS over conventional cardiopulmonary resuscitation, i.e., reduction of in-hospital mortality and improvement of long-term neurological outcomes, and post-arrest survival in H-CA patients [14]. Whereas a study by Belohlavek et al. showed that early intra-arrest transport, ECLS performing and invasive treatment did not significantly raise survival with a neurologically favorable outcome in this group of patients [15]. What is more, the lack of clear evidence of effectiveness in hypothermic patients with preserved circulation has resulted in limiting the indications to use ECMO only in cardiac arrest [7,8,16]. Therefore, patients’ selection for the use of ECMO plays a crucial role in case of accidental hypothermia occurrence [16].

Knowledge about the epidemiology, diagnosis and treatment of accidental hypothermia is gradually evolving, but there are still some gaps in the understanding of this clinical condition. Lack of knowledge, in turn, affects treatment modalities, that, if implemented at the wrong time or in the wrong way, can contribute to increased mortality. Thus, the aim of this review is to discuss the clinical aspects of the management of severely hypothermic patients, with a particular emphasis on the role of ECMO.

## 2. Methods

**Figure 1:**
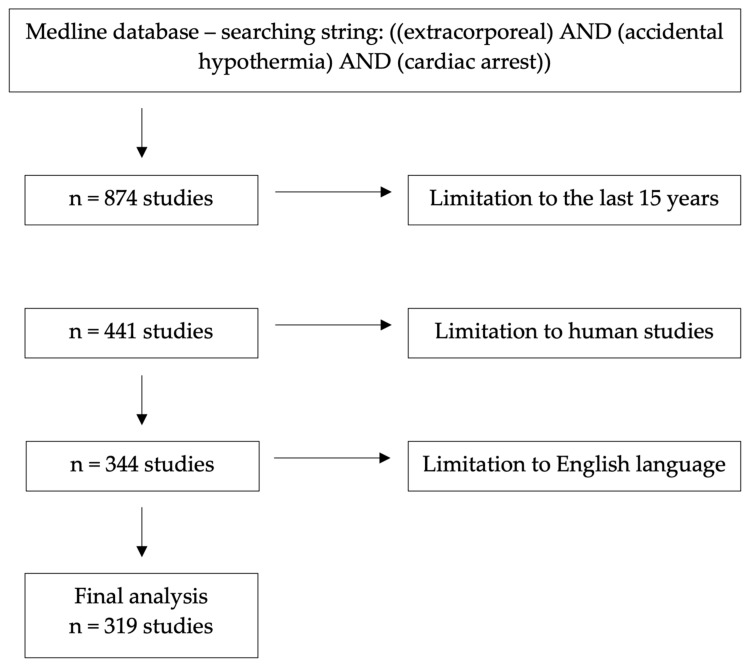
Flowchart of the search strategy.

The search of the Medline database was performed on 20 July 2023 using the advanced search option and the following search keywords: ((extracorporeal) AND (accidental hypothermia) AND (cardiac arrest)). The search was limited to the last 15 years and only human studies written in English were included (Figure 1). It should be highlighted that this study is not a systematic review; thus, the search was simplified. Only reports containing significant information on the topic are referred to in this review. Two researchers independently read all the identified papers.

## 3. Classification of Hypothermia

There are several staging systems for hypothermia. Nevertheless, the most commonly used classifications of accidental hypothermia are a three-stage scale based on Tc measurement (Wilderness Medical Society classification) and a four-stage Swiss Staging System, which is based on clinical symptoms (Table 1) [17,18].

The Swiss Staging System is a useful tool when a reliable Tc measurement is not available [19]. The assessment based on this scale is largely indicative; thus, it may be used for initial diagnosis and implementation of appropriate therapeutic procedures, but not for the final qualification for ECMO therapy.

## 4. Symptoms

The detrimental effect of hypothermia on all systems and organs occurs when Tc already decreases slightly and progresses further with decreasing the temperature. There are 3 phases of pathophysiological alterations observed during Tc decrease. The first response of the human body to an unintended drop of Tc is the activation of the adrenergic system and an increase in the secretion of adrenal and thyroid hormones. This reaction stimulates the cardiovascular and respiratory systems. The second phase is characterized by progressive suppression of the medulla oblongata thermoregulatory centers, which leads to organ dysfunction. Symptoms resembling the state of clinical death are observed in the last, third phase. These pathophysiological changes cause serious disturbances of vital organs and the most characteristic of them are shown in Table 2 [10,20,21].

## 5. In-Hospital Treatment of Accidental Hypothermia and the Role of ECMO

### 5.1. Diagnosis of Accidental Hypothermia

Correct temperature measurement is a key element of proper diagnosis and implementation of optimal therapy in patients with accidental hypothermia. It has been previously thoroughly described how Tc measurement should be correctly performed [22]. A reference technique for Tc measurement is the pulmonary artery temperature reading [23]. However, in clinical settings, the temperature readings in the esophagus are considered to be a gold standard. Tympanic measurement using a thermistor technique is also a reliable option and is an alternative if invasive methods of Tc measurement are impossible to achieve, particularly in patients with preserved circulation. Furthermore, if the urine flow rate is within a normal range, the urinary bladder temperature would closely match the Tc. Temperature-sensing indwelling urinary catheters allow for the continuous drainage of urine and the constant measurement of body temperature. However, if Tc measurement is not available, the estimation of H-CA risk can be made by evaluating the vital signs according to the Swiss Staging System (Table 1).

### 5.2. Initial Management and Qualification for ECMO Support

Measures applied in the pre-hospital management of accidental hypothermia have been exhaustively described by Paal et al. [2]. According to the recent European Resuscitation Council (ERC) Guidelines all hypothermic patients with risk factors, i.e., Tc below 30 °C, ventricular arrythmias and systolic blood pressure below 90 mmHg and those with cardiac arrest, should be transported directly to the centers, where the ECMO rewarming methods are available [8]. However, there are still no clear criteria or algorithms for the qualification of hypothermic patients for ECMO therapy. Taking into account the fact that there are no absolute contraindications for ECMO support, the decision of its use should be balanced between possible complications and potential benefits [10,12].

From a practical point of view, due to frequent Tc measurement unavailability in pre-hospital settings, interviews with eyewitnesses of the hypothermic event are crucial when making decisions. H-CA patients with a cold trunk, a history of cold exposure (indoor or outdoor) preceding asphyxia, with no serious injuries are those who should be rapidly transported to the ECMO centers to increase the likelihood of treatment success. Additionally, the most important principle of the patients’ selection for ECMO therapy is in fact that it should not follow the guidelines for ECMO support for reasons other than accidental hypothermia. Hence, unwitnessed cardiac arrest and conditions such as asystole found at presentation, long or unknown no-flow time (the time from cardiac arrest to cardiopulmonary resuscitation), dilated and fixed pupils, advanced age and end-tidal carbon dioxide below 10 mmHg are not an absolute contraindication to ECMO (Table 3) [10,24].

The evidence supporting traditional triage with Tc and serum potassium level are weak (cut-off level at 30 °C and 7 mmol/L for avalanche victims with cardiac arrest or 12 mmol/L for other remaining cases of accidental hypothermia with circulatory instability) [29]. Extreme hyperkalemia, reflected by an increased serum potassium concentration above the mentioned cut-off values, is regarded as an absolute contraindication to the ECMO, being a marker of cell death and indicating an unfavorable prognosis. In severe hypothermia, potassium concentrations vary, depending on the sampling site and the analytical method used [30]. The kidney and adrenal insufficiency, intoxication or rhabdomyolysis should be excluded in case of extreme hyperkalemia. Moreover, taking into account other factors that may affect survival, the decision-making should never rely on a single clinical marker [8,10]. Thus, the Hypothermia Outcome Prediction After ECLS, which is known as the HOPE score has been proposed in order to establish the survival probabilities of H-CA patients [29]. A calculated probability of survival of ≥10% in adult H-CA patients provides an indication and justification for ECMO support [10,29]. An online calculator of the HOPE score is available at: www.hypothermiascore.org (accessed on 8 September 2023).

VA-ECMO circuit is indicated in H-CA patients with a potential for its reversibility. The beneficial effect of VA-ECMO is not only in rewarming but also in effective circulatory support as VA-ECMO provides extracorporeal gas exchange and adequate organ perfusion [31].

In summary, the following measures should be taken to make the final decision about VA-ECMO support use in H-CA patients:

1. Interview regarding the circumstances of cooling and appropriate Tc measuring.
2. Blood samples from ultrasound-guided puncture of the femoral venous (mainly to assess the serum potassium concentration) [30].
3. Estimation of the survival rate according to the HOPE score [29].
4. Consideration of all possible complications and potential benefits of ECMO therapy.
5. Cardiopulmonary resuscitation should be continued during ECMO cannulation.
6. Further therapy is dependent on the patient’s condition and is the same as for normothermic cardiac arrest patients [10].

### 5.3. ECMO Cannulation Techniques and Complications

Cannulation in severely H-CA patients has different specificity compared to normothermic subjects. Depending on the patient’s characteristics and the skills of the medical staff, the cannulation of the peripheral vein and artery is performed by the following methods [10,31]:

1. Percutaneous modified Seldinger technique, which is associated with a lower risk of bleeding and infection. Rapid implementation is a benefit of this method, but it can be difficult to perform in hypothermic patients with prolonged cardiopulmonary resuscitation. This technique should be performed using ultrasound guidance.
2. By using the surgical open cutdown technique, when percutaneously cannulation is not possible. This method is preferred in many centers.
3. Combination of the surgical open cutdown and Seldinger techniques.
4. During cannulation chest compressions should be continued with the best achievable quality until sufficient ECMO flow is achieved. Chest compressions can then be terminated [10].

Similarly, to the occurrence of problems and pitfalls in the qualification procedure for the ECMO, difficulties are also observed in cannulation. One of the most serious complications is associated with inadequate distal leg perfusion, which is related to the femoral artery cannulation and results in lower extremity ischemia (about 20% of ECMO patients) [31,32]. Hence, a separate perfusion line is usually placed in the distal superficial femoral artery by direct cutdown for retrograde perfusion. This catheter is connected to the side port of the arterial cannula using a 6–8 French extension tubing with an intervening three-way stopcock [31]. Notably, Takayama et al. showed that insertion of the smaller arterial cannulas is associated with lower arterial complication rates compared to the larger ones, while clinical support was comparable [33]. Despite this, limb ischemia is a common cause of premature ECMO system removal [34].

### 5.4. ECMO Circuit and Patient-Related Management

The success of the ECMO therapy depends also on proper surveillance during extracorporeal gas exchange and perfusion. The most important principles of circuit and patient-related management in H-CA patients are listed below.

#### 5.4.1. Blood Flow, Oxygenation and Hemodynamics Support

According to the 2017 Extracorporeal Life Support Organization (ELSO) guidelines, to assure optimal systematic perfusion, the blood flow should be between 3 and 4 L/min in adult patients [35]. Blood flow along with vascular resistance have an impact on hemodynamics during ECMO treatment. The mean arterial pressure goal ranges between 50 and 70 mmHg. Hypovolemia is frequently observed in hypothermic patients as a result of endothelium damage and fluid shifts. The most reliable indicator of adequate flow seems to be venous oxygen saturation over 70% [35]. Of note, decreased oxygen demand observed in hypothermia might be a cause of venous oxygen saturation elevation [10].

Refractory ventricular fibrillation may occur during rewarming, which is resistant to the standard therapy until Tc > 30 °C is reached. In case of bradycardia and hemodynamic instability after achieving normothermia, when pharmacological treatment is ineffective, internal cardiac pacing should be considered [10].

The ECMO support should be completed when normothermia with sufficient hemodynamic stability is achieved or persistent asystole/irreversible ventricular fibrillation occurs, despite normothermia [36]. Complications, such as severe respiratory failure, septic shock with high vasopressors demand and post-resuscitation chest trauma, may delay weaning. With echocardiographic evidence of cardiac recovery, ECMO blood flow should be reduced gradually to a minimum rate of 1.2 to 1.5 L/min. The criteria of the weaning from ECMO are same for hypothermic as well as normothermic patients, i.e., left ventricle ejection fraction ≥ 25%, aortic velocity-time interval ≥ 12 cm on minimal ECMO support settings (1.5 L/min), mitral annulus peak systolic velocity ≥ 6 cm/s, no major valvular pathologies, mean blood pressure > 60 mmHg and central venous pressure < 18 mmHg [37].

#### 5.4.2. Temperature Management

Continuous Tc measurement should be held in two sites simultaneously [9]. Several sites are possible, such as the esophagus, urinary bladder, rectum, tympanic membrane, pulmonary artery or venous inflow [22]. However, ERC strongly recommend esophageal measurement [8]. The target position of the tip of the probe is located in the lower third of the esophagus. However, the esophageal temperature may not correspond to the Tc, which might be caused by warm blood outflow in the vicinity of the tip of the esophageal probe. According to the ERC guidelines, the target Tc during ECMO support is ≥32 °C.

The rewarming rate is calculated as a change of Tc per unit of time during ECMO therapy (°C/h). The optimal rewarming rate is still unknown for hypothermic patients but is usually targeted to ≤5 °C/h [38]. Faster rewarming rates until the return of spontaneous circulation may be recommended, but 10 °C/h should not be exceeded, followed by slower rewarming of about 1–2 °C/h. The heat exchanger should not be set above 37 °C to prevent brain tissue overheating [35]. Normothermia should be achieved within 24 h after the return of spontaneous circulation. When continuous renal replacement therapy is carried out simultaneously, the temperature should be set to 37–38 °C.

#### 5.4.3. Anticoagulation and Hemostasis Disturbances

ECMO protocols routinely include the use of heparin in order to counteract the development of the prothrombotic state and decrease the risk of pump malfunction, oxygenator failure and thromboembolic events, including stroke or pulmonary embolism [38,39,40,41]. Bolus of unfractionated heparin should be administered (50–100 units per kg) at the time of cannulation and then by continuous infusion [35,39]. An activated clotting time (ACT) targeted at 160–220 s is recommended [39]. The benefit of the ACT is rapid assessment of the haemostasis at the patients’ bedside. However, several factors can affect ACT, including hypothermia itself [41,42]. Recent reports have analyzed the alternative anticoagulation strategies, which include bivalirudin or nafamostat mesilate use instead of heparin [43,44]. This issue requires further studies.

Potentially life-threating bleeding is recognised as a complication during VA-ECMO, but data on the optimal strategy for anticoagulant therapy are limited and largely based on experts’ opinion [32]. However, to avoid systemic anticoagulation and to reduce the risk of bleeding, heparin-coated ECMO systems and high-flow techniques are used [12,45]. All lesions found in computed tomography, like intracranial bleeding, brain masses or skull fractures, should be consulted with neurosurgeons to establish the risk-benefit ratio of ECMO support. On the other hand, low and nonpulsatile blood flow is associated with the prothrombotic clot phenotype and, thus, predisposes to thrombosis [46,47,48], which could be another complication of the ECMO support. The observed hypercoagulability is also a result of ECMO biomaterial-mediated activation of the coagulation and inflammation pathways and increased platelet activation [47,48]. Of note, echocardiography examination may mistakenly indicate intracardiac coagulation, but in fact, this phenomenon is associated with increased blood echogenicity and slow flow induced by hypothermia [10]. Systemic anticoagulation may be waived within the first 24 h in case of coagulopathy induced by the ECMO system, bleeding or high risk of its occurrence [49].

#### 5.4.4. Ventilation

The lung protective ventilation strategy, based on the smallest possible volume and driving pressure, includes a tidal volume of 6 to 8 mL/kg ideal body weight with a maximum positive end-expiratory pressure of 10 cmH_2_O or a plateau pressure of 20–25 cmH_2_O [35,50]. The latter setting should be adjusted according to cardiorespiratory function. An initial ventilator setting of FiO_2_ is 1.0 [10]. To avoid the risk of alveolar damage and hyperoxia, FiO_2_ should be reduced to the lowest possible value. Moreover, management should focus on pulmonary edema prevention [35].

## 6. Effectiveness and Prognosis after ECMO Therapy in Accidental Hypothermia

In VA-ECMO treated, normothermic patients’ mortality is estimated at 60 to 80% in case of cardiogenic shock and cardiac arrest [51]. However, in accidental hypothermia, the overall mortality is difficult to estimate due to the heterogeneity of the patients included in different analyses. An analysis of 44 observational studies and 40 case reports, comprising 658 patients with accidental hypothermia treated with ECLS, revealed an overall survival rate with a good neurologic outcome of 40.3% [52]. In patients with H-CA, survival at 28 days was significantly higher in the ECMO group (58.3%) than in the non-ECMO group (21.2%) in a recently published Japanese ICE-CRASH study [53]. In the HOPE derivation and validation studies survival rate of 37–42% was reported [29,54]. Notably, in both studies by Pasquier et al., only cardiac arrest patients were included. It is worth emphasizing the high percentage of patients treated with ECLS who survive in good neurological condition. This may be due to the neuroprotective effect of hypothermia, but also to the better organ blood flow obtained with ECMO support. When comparing the effectiveness of conventional cardiopulmonary resuscitation vs. extracorporeal cardiopulmonary resuscitation in normothermia, better neurologic results are observed after extracorporeal methods of treatment [55,56].

Several demographic and clinical variables have an impact on the effectiveness of ECMO therapy in H-CA patients. Some of them, along with the literature review, are shown in Table 4.

The prognostic factors associated with good patient outcomes and availability at hospital admission were described and validated in the HOPE studies [references—see Table 4]. The HOPE score consists of 6 variables:

- Age;
- Sex;
- Tc;
- Serum potassium;
- Presence of asphyxia;
- Duration of cardiopulmonary resuscitation.

Despite these well-established factors, the estimation of the final success of this treatment is still challenging. There are not any identified parameters measured during ECMO therapy that could predict the final results of this treatment. A recent study showed that higher lactate concentrations during VA-ECMO therapy were associated with a poor prognosis for hypothermic patients undergoing ECMO rewarming [58]. However, this observation requires further research.

To sum up, there are still issues that need further investigation, such as the development of protocols in each phase of treatment of H-CA patients, from pre-hospital triage, transport, qualification and identification of those patients, in whom optimal results might be obtained. There is also a lack of unambiguous principles of safe anticoagulation and vasopressor therapy during ECMO. Last but not least, the optimal rewarming rate should be clearly established.

## 7. Conclusions

VA-ECMO, as the most powerful method of providing oxygenated blood to vital organs during cardiac arrest, may significantly improve prognosis in H-CA patients. However, the key to success is the careful selection of patients in whom optimal treatment results can be obtained. It has to be emphasized that eligibility criteria for ECMO support are different in normothermic and hypothermic cardiac arrest patients. Further research is needed to better exploit the neuroprotective effect of hypothermia, to better identify the special risk groups and to improve the results of ECMO support in patients with hypothermic cardiac arrest.

## Figures and Tables

**Table 1:** Summary of the two main clinical staging systems for accidental hypothermia: Wilderness Medical Society classification and the Revised Swiss Staging System.

WMS			Revised Swiss System		
Category	Clinical Findings	Estimated Tc (°C)	Stage	AVPU Level of Consciousness	Risk of Hypothermic CA
Mild	Normal mental status, shivering, but not functioning normally and unable to care for self	35–32	Stage 1	“Alert”	Low
Moderate	Abnormal mental status with shivering, or abnormal mental status without shivering, but conscious	32–28	Stage 2	“Verbal”	Moderate
Severe/profound	Unconscious	<28	Stage 3	“Painful’’ or “Unconscious” and vital signs present	High
			Stage 4	“Unconscious” and no detectable vital signs	Hypothermic CA

Abbreviation: AVPU: Alert, Voice, Pain, Unresponsive; CA: cardiac arrest; Tc: core temperature.

**Table 2:** Pathophysiological alterations and clinical manifestations in relation to stage of hypothermia.

Physiological Changes and Clinical Symptoms				
	Mild (35–32 °C)	Moderate (32–28 °C)		Severe (<28 °C)
Metabolism and hormones	- ↑ secretion of catecholamines and thyroid hormones: Hyperglycemia Shivering ↑ oxygen consumption - ↓ cerebral metabolism (neuroprotection effect)	- ↓ metabolism rate - Hypoglycemia/hyperglycemia - Loss of shivering - ↓ oxygen consumption		- ↓ hormonal secretion and peripheral adrenaline activity < 20 °C
Cardiovascular system	- Tachycardia - Supraventricular arrythmias - ↑ CO and MAP - Prolonged PR and QT intervals	- Progressive bradycardia (resistant to atropine) - Supraventricular and ventricular arrythmias - ↓ CO - Elevation or depression of ST-segment and T wave - Osborn wave (80% cases < 30 °C of Tc)		- Progressive bradycardia (resistant to atropine) - Ventricular arrythmias - Asystole < 20 °C - ↓ CO and MAP
Central nervous system	- Confusion - Amnesia - Ataxia - Dysarthria	- Progressive ↓ of consciousness - Dilated pupils - Hallucinations - ↓ EEG activity		- Coma - Nonreactive pupils - Global loss of reflex - Isoelectric line in EEG
Respiratory system	- Tachypnoea - ↑ MV - Risk of respiratory alkalosis	- Progressive bradypnea - ↓ MV - ↑ physiological respiratory dead space due to bronchodilation		- Apnea < 24 °C - ARDS - Pulmonary edema - Stiff chest (chest compressions may be harder than normal)
Hemostasis	Coagulopathy, as a result of: - ↓ platelet count and their activity - ↓ activity of coagulation factors and thrombin - Massive activation of fibrinolysis < 20 °C - Total inhibition of the hemostasis < 16 °C			
	Or hypercoagulability - ↑ risk of DIC - ↑ of hematocrit by 2% with every fall in 1 °C of Tc below 34 °C			
Acid-base balance	Metabolic alkalosis		Lactic acidosis	
Electrolyte disturbances	Hyper- or hypokalemia			

Abbreviations: ARDS: acute respiratory distress syndrome; CO: cardiac output; DIC: disseminated intravascular coagulation; EEG: electroencephalogram; MAP: mean arterial pressure; MV: minute ventilation; Tc: core temperature. ↑ denotes increase and ↓ denotes decrease.

**Table 3:** Examples of inclusion criteria for extracorporeal membrane oxygenation (ECMO) in normothermic vs. hypothermic cardiac arrest patients.

CA Patients	
Normothermia [25]	Hypothermia
Age < 70 years	No age limitations [26]
Witnessed arrest	Witnessed and unwitnessed arrest [27] or Cardiocirculatory instability with Tc of ≤28 °C [13]
Arrest to first CPR (“no-flow interval”) <5 min (i.e., bystander CPR)	Possible in patients with unwitnessed CA and long no-flow time with a good therapeutic effect [28]
Initial cardiac rhythm of VF/pVT/PEA	Initial cardiac rhythm of VF/pVT/PEA or asystole [27]
Arrest to ECMO flow <60 min “low flow interval” *	Usually longer than 60 min [10]
ETCO_2_ > 10 mm Hg (1.3 kPa) during CPR before cannulation for ECMO	Low ETCO_2_ should not be used in hypothermic CA as exclusion criteria [24]
Intermittent ROSC or recurrent VF	Possible no ROSC or persistent VF [10]
“Signs of life” during conventional CPR may be a positive predictive factor for survival	Fixed and dilated pupils or no signs of life should not be used in hypothermic CA as exclusion criteria [10]
The absence of previously known life-limiting comorbidities (e.g., end-stage heart failure/chronic obstructive pulmonary disease/end-stage renal failure/liver failure/terminal illness) and consistent with the patient’s goals of care	Compatible with normothermic CA
No known aortic valve incompetence (>mild aortic valve incompetence should be excluded)	Compatible with normothermic CA

* Unless other favorable prognostic features are present: e.g., periods of intermittent ROSC/hypothermia prearrest/young age/signs of life during CPR. Abbreviations: CA: cardiac arrest; CPR: cardiopulmonary resuscitation; ECMO: extracorporeal membrane oxygenation; ECPR: extracorporeal cardiopulmonary resuscitation; ETCO_2_: end-tidal carbon dioxide; PEA: pulseless electrical activity; pVT: pulseless ventricular fibrillation; VF: ventricular fibrillation; ROSC: return of spontaneous circulation.

**Table 4:** Prognostic impact of demographic and clinical factors on the effectiveness of extracorporeal membrane oxygenation (ECMO) therapy in hypothermic out-of-hospital cardiac arrest patients.

Variable	Favorable Factors	Adverse Factors
Serum potassium concentration	Lower level; ≤8 mmol/L [29,57]	Severe hyperkalemia, frequently proceed by hypokalemia; the cut-off level at 12 mmol/L for accidental hypothermia and 7 mmol/L for hypothermia due to avalanche burial in-hospital triage [56]
Sex	Female [29,52,57]	Male [57]
Initial lactate	Lower concentration: <11.9 mmol/L was a predictor of a good prognosis [28,58]	Higher concentration: >16.3 mmol/L was associated with worse neurologic outcomes, >21 mmol/L resulted in premature end of ECMO therapy [58,59,60]
Initial pH	Higher blood pH [56,58,61]	Lower blood pH; pH < 7.35 within 24 h during ECMO therapy [28,57]
Initial Tc	Less drop of Tc [28]	The calculated cut-off value for Tc was 30 °C for in-hospital triage [62]
Asphyxia	Non-asphyxia-related mechanism of cooling [28]	Asphyxia, especially with hyperkalemia [29,52]
Time of resuscitation	Shorter time [28]	Longer time; CPR prior to ECMO > 40 min [63]
Infusion fluids and diuresis	Fluid balance < 8500 mL after 24 h and higher total urine output [64]	Higher fluid balance; more common occurrence of anuric and oliguric patients in the non-survival group [64]

Abbreviations: CPR: cardiopulmonary resuscitation; ECMO: extrapulmonary membrane oxygenation; pH: potential hydrogen; Tc: core temperature.

## Data Availability

Not applicable.
